# Supplementary material for: Sweetpotato-based infant foods produce porridge with lower viscosity and aflatoxin level than cereal-based complementary blends
Source: PLoS One. 2022 Oct 13;17(10):e0275593. doi: 10.1371/journal.pone.0275593 (PMC9560132; doi:10.1371/journal.pone.0275593)
Supplement: Attachment — Submitted filename: Rebuttal.docx [file pone.0275593.s001.docx]

**Response to queries in italics: Reference to revised manuscript with track changes**

Reviewer reports:

**Reviewer #1:**

Although the aims of the study are good, it was planned and carried out very superficially. It has been seen that the written language, sample, analyzes and methods of construction are not sufficient. Samples are not well explained, not understandable. It is thought that measuring each of the samples in the viscosity study by bringing them to the same dry matter significantly affects the difference between the samples. It is thought that the evaluation made in this way will not give correct results.

*Your comments are very much appreciated. However, the samples were existing proprietary products (lines 107 – 108); thus, their brand names were undisclosed for ethical reasons. Therefore, the best way to describe these samples without disclosing brand names was to indicate their constituent ingredients, as captured in lines 107 – 108. Regarding analyses and methods, some revisions have been made (lines 103, 105 – 106, 131 – 135).*

*Although what you have suggested is right for getting the actual viscosities of the samples. It is required, in rheological analyses such as visco-analysis, that samples are corrected to the same dry matter to avoid wrong comparisons as a result of varied initial moisture contents. With the same dry matter content, the viscosity of the samples can best be compared, hence the reason to correct the samples to the same dry matter content in this study.*

**Reviewer #2: PONE-D-22-17748-peer-review-v1.comments**

Comment 1: The research is quite innovative and interesting, with clearly established objectives and conclusions.

*Thank you very much for your kind remarks.*

Comment 2: Abstract: The following line is little bit confusing, it is recommended to rephrase it to make it more clear and easily understandable: 'However, the protein content in the porridges on an as-would-be-eaten basis was about 6% lower.'

*Thank you for your comment. The statement has been rephrased: ' However, the protein content in the porridges on an as-would-be-eaten basis was about 6% lower than the as-is basis value.'*

Comment 3: Abstract: Have you mentioned the amount and the desirable water content for proper mixing of the blends?

*No, please, reason being the word limit for the abstract. However, it has been stated in the materials and methods section (lines 139 – 142).*

Comment 4: 62: Is not 10 ppb really high even to be marked as maximum level for infant cereals, when we are talking about the acceptable levels as 0.18-0.39 ppb?

*Yes, you are very right. The statement has been revised as "To curb the negative effect of aflatoxin, its ingestion from food should be kept as low as possible (19)."*

Comment 5: Line No. 109: Did you measures moisture and protein content only in nutritional parameters? If so, what about the determination of other nutritional contents like minerals, fiber and vitamins?

*Yes, please. Moisture and protein contents were the only parameters measured. The study did not include other nutritional properties due to financial constraints, which was captured as a limitation (lines 248 – 250).*

Comment 6: Line No. 150: Have you mentioned anywhere about total number of samples or treatments that you have used?

*Yes, please. The total number of samples (24) was mentioned in line 97.*

Comment 7: Line No. 223: In my opinion this level of alfatoxin as reported from the results here are high for infant consumption. Please add justification.

*You are right. The aflatoxin level reported is for infant consumption. Lines 243 – 247 underscores the importance that the complementary blends assessed were not safe for infant consumption as all of them had toxin level above the permissible limit for infants as per Switzerland, Netherlands, and European standards and the need for stricter regulatory measures.*

Comment 8: Line No. Conclusion: Overall the study is good, but in this type of study a small animal based efficacy investigation should also be included as to ensure pros and cons of the product developed. Without having its safety confirmation we are unable to recommend such innovative idea.

*Your comment is highly appreciated. However, the products used for this study were on the market, and were not developed by the author. Thus, the study could not include any animal-based investigations.*

**Reviewer #3:**

The research article "Sweetpotato-based infant food as an alternative complementary formulation" by Francis seeks to describe the combination of different ingredients and their properties and product studies (protein and viscosity).

The article needs extensive corrections.

*Your comment is greatly appreciated. Some revisions have been made to that effect.*

I think the author should change the title it did not match with the content.

*Thank you very much. The title has been changed as suggested to "Sweetpotato-based infant foods have lower viscous porridge and total aflatoxins level than cereal-based complementary blends" in lines 1 – 2.*

Comment 1: First, the English language has to be corrected, the authors use too much every-day spoken English, too much for a scientific publication.

*Thank you for your comment. The safety and quality of complementary foods are critical; thus, there is the need to present it in such a way that the general populace can read and understand the manuscript. I am also finding it difficult where the "every-day spoken English" phrases are in the manuscript.*

Comment 2: Authors should add the descriptive sensory analysis or 9-point hedonic scale and shelf life of porridge.

*The author appreciates your comment. However, the samples used in this study were existing products on the market and not developed by the author. Hence, sensory analysis and shelf-life studies were not relevant. These are done during the product development phase.*

Comment 3: The author should add the physico-chemical properties of multigrain porridge such as fat, crude fiber, ash, and carbohydrates.

*Thank you. The author would have loved to determine the aforementioned physico-chemical properties. However, the study did not include these properties due to financial constraints, as indicated in lines 248 – 250. Moreover, being proprietary products, the nutritional information was on the package. The cereal-only sample, particularly from white-coloured maize, has been reported in previous work to be nutritionally inappropriate for complementary feeding.*

*Amagloh, F. K., Weber, J. L., Brough, L., Hardacre, A., Mutukumira, A. N., & Coad, J. (2012). Complementary food blends and malnutrition among infants in Ghana–A review and a proposed solution. Scientific Research and Essays, 7(9), 972-988. doi:10.5897/SRE11.1362*

Comment 4: Author should check the dough's rheological properties which are prepared from orange maize.

*The viscosity measured for the (fermented) orange maize is one of the rheological properties.*

Comment 3: The abbreviation CHE have no sense in line number 104-105, the abbreviation details should be added in line number 101-104 (The ingredients of the control formulation included whole corn flour (CHE indicated on the package), skimmed milk, sucrose, palm olein, acidity regulator, calcium carbonate, vitamins, ferrous fumarate, vanillin, zinc sulphate, Bifidus culture, and potassium iodide.)

*Comment well noted and the revision has been done appropriately. Lines 103, 105 & 106.*

Comment 6: In line number 114 the author should clearly add which type of dry process and instrument were used for drying at 108°C overnight.

*Thank you. The instrument used was a Thermo Scientific Heratherm oven. This has now been added to the revised manuscript (lines 115 – 116) as suggested.*

Comment 7: Rewrite the viscosity measurement protocol from line number 124-142.

*Thank you. Please, some revisions have been made as suggested, as captured in lines 131 – 135, 138 – 139.*

Comment 8: In Table 1 no need for the repetition of (n=1), you can add it at end of the table

Example: Data are presented as mean ± SD (n = 3)

a-nMeans with the same superscript in a column do not vary significantly (p<0.05) from each other

In table 1 author adds the value please justify then and explain in paragraph also. Or the author removes those values.

*Your comments are well noted. Please, revisions have been made to that effect. Lines 223-225.*

Comment 9: The author checks the result discussion and conclusion part.

*Thank you very much. The author has re-checked the abovementioned areas as suggested.*
